# The GRK2 Overexpression Is a Primary Hallmark of Mitochondrial Lesions during Early Alzheimer Disease

**DOI:** 10.1155/2009/327360

**Published:** 2010-03-03

**Authors:** Mark E. Obrenovich, Hector H. Palacios, Eldar Gasimov, Jerzy Leszek, Gjumrakch Aliev

**Affiliations:** ^1^Department of Pathology, Case Western Reserve University, Cleveland, OH 44106, USA; ^2^Department of Biology, College of Sciences, University of Texas at San Antonio, San Antonio, TX 78249-1664, USA; ^3^Electron Microscopy Research Center, College of Sciences, University of Texas at San Antonio, San Antonio, TX 78249-1664, USA; ^4^Department of Cytology, Histology and Embryology, Azerbaijan Medical University, Baku AZ10-25, Azerbaijan; ^5^Department of Psychiatry, Wroclaw Medical University, Wroclaw 50-229, Poland; ^6^School of Health Science and Healthcare Administration, University of Atlanta, Atlanta, GA 30360, USA

## Abstract

Increasing evidence points to vascular damage as an early contributor to the development of two leading causes of age-associated dementia, namely Alzheimer disease (AD) and AD-like pathology such as stroke. This review focuses on the role of G protein-coupled receptor kinases (GRKs) as they relate to dementia and how the cardio and cerebrovasculature is involved in AD pathogenesis. The exploration of GRKs in AD pathogenesis may help bridge gaps in our understanding of the heart-brain connection in relation to neurovisceral damage and vascular complications of AD. The a priori basis for this inquiry stems from the fact that kinases of this family regulate numerous receptor functions in the brain, myocardium and elsewhere. The aim of this review is to discuss the finding of GRK2 overexpression in the context of early AD pathogenesis. Also, we consider the consequences for this overexpression as a loss of G-protein coupled receptor (GPCR) regulation, as well as suggest a potential role for GPCRs and GRKs in a unifying theory of AD pathogenesis through the cerebrovasculature. Finally, we synthesize this newer information in an attempt to put it into context with GRKs as regulators of cellular function, which makes these proteins potential diagnostic and therapeutic targets for future pharmacological intervention.

## 1. Introduction

G protein-coupled receptor kinases (GRKs), such as GRK2, are cytosolic proteins that contribute to the adaptation of the heptahelical G protein-coupled receptors (GPCRs) and to the regulation of their downstream signals. GPCRs mediate the action of messengers that are key modulators of cardiac and vascular cell function [1]. A family of seven mammalian serine/threonine protein GRKs have been identified. GRK2 and 3 form the second subfamily, namely, *β*-adrenergic receptor kinase (*β*ARK), whose members can phosphorylate and regulate agonist-occupied or constitutively active GPCRs [2]. When recruited to the cell membrane, homology domains of GRK2 modulate the simultaneous inhibition of signaling by G-alpha, G-beta, and G-gamma subunits. Recent studies suggest that GRKs, particularly GRK2, may have more diverse protein/protein cellular interactions because of a consensus caveolin binding motif within the pleckstrin homology domain of GRK2 [3]. 

Normal aging and sporadic late-onset AD have many features in common. AD-like symptoms only appear to manifest when certain quantitative levels of damage occur [4, 5]. This damage includes metabolic and oxidative stress, as well as those factors associated with impaired cerebral perfusion, which all are attributed to risk. Examples of impaired perfusion include cardiovascular and cerebrovascular diseases, hypo/hypertension, and stroke, which impair the body's ability to adequately cope with further insults [5, 6]. We speculate that an imbalance in the activity of nitric oxide synthase (NOS) isoforms, endothelin-1 (ET-1), and oxidative stress, as evidenced by several biomarkers of this damage, along with mitochondrial DNA (mtDNA) aberrations and mitochondrial enzyme imbalance in vascular wall cells and neurons, leads to inadequate antioxidant response [4]. An adequate antioxidant reserve capacity is needed to sufficiently abate metabolic and oxidative insults, which are two key initial features in the brains of stroke and AD patients [7]. We further hypothesize that GRK2 plays a role in these deleterious processes [8]. 

Under conditions associated with advanced aging, any imbalance in the activity of NOS isoforms, ET-1, and oxidative stress can lead to a potential and very destructive positive feedback loop [9–11], in which increased levels of reactive oxygen species (ROS) interfere with NO function, lead to endothelial relaxation by reducing its bioavailability (through ROS scavenging), increase the amount of oxidative stress levels through the production of peroxynitrite, impair endothelial barrier function, promote leukocyte adhesion, and induce alterations in normal vascular function, which further decreases cerebral blood flow (CBF) [11]. It appears that transient GRK2 activity correlates with compensatory changes to oxidative stress and arterial occlusion, including changes in endothelin-1 (ET-1) expression [4, 10]. Although we are aware that correlation does not necessarily imply causation, we are equally cognizant of the axiom necessitating correlation in order for causation to be supported. With this in mind, we investigate what role GRK2 may play in the early pathogenesis of dementia and determined the cellular, subcellular, and ultrastructural distribution and localization of GRK2 immunoreactivity in cases of human AD as well as in a mammalian model of chronic brain hypoperfusion (CBH), as first seen with light microscopy and confirmed by western blotting [8].

Additionally, the involvement of CBH and physical distortion of the surrounding tissue exacerbate this imbalance and more than likely contribute to the collapse of postischemic/hypoxic vessels. Sustained hypoperfusion and oxidative stress, both primary features of aged brain tissue during the prodromal stages of AD [6, 12, 13], also may stimulate the expression of various NOS species and subsequent ET-1 in brain cells and probably increase the accumulation of oxidative stress products, thereby contributing to blood-brain barrier (BBB) breakdown, increased NO, and peroxynitrite production and resulting in brain parenchymal cell damage (for a more in-depth discussion regarding the interactions of each of these factors please see our previous works [10, 14]). These findings raise questions regarding the direct relationship between oxidative stress, energy failure (e.g., mitochondrial lesions) or metabolic insufficiency, neuronal and vascular damage, BBB breakdown, and A*β* deposition during the maturation of AD-like pathology [4, 10].

Increasing evidence for the roles of GRKs and angiotensin 1 and 2 (AT_1_ and AT_2_) in hypertension, stroke, and heart disease with association between these receptors/ligands in heart disease and AD [15], as well as early amyloid-*β* (A*β*) accumulation in vitro [16] and our in vivo work with models of hypoperfusion [8] prompts further consideration of AD and AD-like pathology in terms of possible inclusion and classification as disorders of the cerebrovasculature, because they involve common receptor types. Our in vivo findings demonstrated the early involvement of this kinase in both cerebrovascular ischemia and AD [8]. During ischemic injury and in the vulnerable neurons of AD patients, we found increased GRK2 immunoreactivity. Therefore, cellular and subcellular investigations into the mechanisms preceding A*β* deposition and progression, as well as the possible accelerating effects of environmental factors such as chronic hypoxia/reperfusion, were crucial to understanding of events that precede amyloid deposition and which may lead to insights into new pharmacological treatments of AD [8, 17].

## 2. General Features of GRKs

GRK function and interaction are complicated and its importance compels increasing research interest. GRKs function to regulate receptor trafficking of GPCRs and, thus, dictate the appropriate signal magnitude and specificity for the downstream signal events from diverse extracellular stimuli, controlling a vast number of physiological processes. GRKs regulate numerous receptor functions in both the brain and myocardium [18] and its actions are more functionally diverse than previously thought. General features of GRK interaction with GPCRs involve complex regulatory mechanisms that modulate receptor responsiveness and underlie signal integration and desensitization [19]. GRKs are members of a multigene family, which are classified into three subfamilies. GRK2 and 3 form the second subfamily (beta-adrenergic receptor kinase (beta-ARK) subfamily), which phosphorylate and regulate agonist-occupied or constitutively active GPCRs. BetaARK1 (also known as GRK2) is the most abundant GRK in the heart, and it is increased in several cardiovascular diseases associated with impaired cardiac signaling and function, suggesting that this protein could have pathophysiological relevance in the setting of heart failure. 

GRKs phosphorylate PAK, MEKK/Raf, MEK, MAPK, SAP/JNK, p38, Arrestins, beta2-adrenergic receptor, and other proteins. GRKs phosphorylate and recruit beta-arrestin to the receptors in order to attenuate receptor desensitization and initiate internalization of G protein-coupled receptors by phosphorylating serine and threonine residues and trigger arrestin binding, which can then be sorted to endosomes and lysosomes. Further, covalent modification of GPCRs with ubiquitin serves as a signal for internalization as well. Failures in ubiquitin-mediated proteolysis may explain the accumulation of GRKs in AD and hypoperfusion, where posttranslational modification of critical lysine residues may impair proteolysis. Many receptors for neurotransmitters and hormones rely upon members of the Gq-alpha family of heterotrimeric G proteins because they directly link receptors to the activation of PLC-beta isoforms, which, in turn, stimulate inositol lipid, calcium, and PKC signaling. Hubbard and Hepler demonstrated that Gq-alpha, G11-alpha, G14-alpha, and G15/16-alpha regulate both overlapping and distinct signaling pathways independent of inositol lipid signaling, while also showing the inhibition of Gq-alpha-dependent PLC-beta1 activity by PKG and PKA when mediated by stimulatory phosphorylation of RGS4 and GRK2 [20].

GRKs critically regulate beta-arrestin signaling via receptor phosphorylation and the triggering of desensitization. The beta-arrestins play a crucial role in regulating the responsiveness of multiple GPCRs [19]. The molecular mechanisms of desensitization are quite complex and have been investigated largely with the beta2-adrenergic receptor (beta2AR) used as the main model system. Recent data from Mayor and colleagues indicate that, besides the uncoupling function, GRK2 and beta-arrestin also directly participate in beta2AR sequestration, thus providing the trigger for its resensitization. This is followed by binding of uncoupling proteins (arrestins) and transient receptor internalization, which plays a key role in resensitizing GPCR by allowing its dephosphorylation and recycling [19]. A detailed knowledge of the role of GRKs and arrestins in betaAR internalization would make their physiologic role in the modulation of cellular responses to messengers better understood and is much too complex to address in this review. It has been shown that stimulated *β*1-AR can recruit GRK to the membrane. Either *β*-AR subtype's interaction with GRKs is via the intracellular loops and at the c-terminus of the receptors.

Recent work has revealed potential phosphorylation-independent regulation of GPCRs by GRK2 and GRK3 [21]. Further, GRKs themselves may be regulated by caveolin [3]. Nevertheless, reduced expression of GRK and beta-arrestins leads to supersensitization of GPCRs and increases the response to neuropeptides, neurotransmitters, chemokines, and many other molecules. Thus, overexpression of these GRKs could serve as a protective or compensatory response to these stressors and chronic stress conditions involving excitotoxicity. Further, GRKs together with A-kinase anchoring proteins (AKAPs) assemble several cAMP effectors, including target protein kinase A (PKA) for cAMP signaling to the cytoplasmic surface of mitochondria, and stimulate PKA-dependent phosphorylation of the proapoptotic protein BAD, which inhibits release of cytochrome *c* from mitochondria, and protect cells from apoptosis [22]. 

## 3. Expression and Localization of GRKs

The various GRK subtypes differ in their localization, regulation, and mode of action. Many GRKs are highly expressed in the heart, brain, and other tissues as shown in the rat and hamster [23], where they regulate numerous receptor functions in the brain and myocardium [24]. Desensitization and resensitization of a wide variety of GPCRs are processes involved in numerous brain functions and GRK2 expression is increased in the developing rat brain, which is consistent with an involvement in brain maturation processes [25]. The expression in the developing brain and in AD is another hallmark of AD, which is characterized by ectopic expression of a multitude of cell cycle markers and proteins that are involved in cell division and development, which we propose as being an apparent recapitulation of ontogeny in this disease [26]. These same analogies have been considered in the parallels observed between AD and cancer and we propose that they are examples of embryological parallelism in both diseases [27]. In the rat brain, mRNA expression pattern of GRKs family of proteins (GRK2, GRK3, GRK4, and GRK6) was widely distributed and had nearly the same expression pattern, although GRK3 generally was expressed more weakly than GRK2 in most tissues. 

GRK2 has been well characterized in the heart, where the onset of congestive heart failure (CHF) is associated with characteristic changes in myocardial expression of GRK2 and is known to significantly contribute to myocardial regulation and function in the failing heart [28]. Signaling through cardiac *β*-adrenergic receptors (*β*ARs) is significantly impaired in many cardiovascular disorders, including CHF. Chronic heart failure leads to upregulation of GRK2, both in cardiac myocytes and in adrenal chromaffin cells, which results in increased phosphorylation and desensitization of betaARs. Further, elevated levels of GRK2 mRNA and GRK2 activity have been reported in human left ventricle explants from heart failure patients [29]. In the heart, *β*ARs control numerous trophic responses to the catecholamine neurotransmitters, norepinephrine, and epinephrine. Heart failure onset is characterized by reduced responsiveness to *β*-adrenoreceptor in cardiac tissues [30] and by changes in the expression of GRK2 or *β*-adrenoreceptor kinase1 (bARK1) and signaling of these receptors through the Gs protein-AC-PKA signaling pathway [31]. When *β*-adrenoreceptor responsiveness was examined in a completely developed reperfused myocardial infarction model, higher levels of tissue catecholamines and GRK2 were observed in the ischemic epicardium, leading to sympathetic overstimulation of the failing heart [32]. It was found that the density of the *β*-adrenoreceptor in the viable ischemic regions can be modified by GRK2 and catecholamines. Conversely, cardiopulmonary intervention was found to decrease GRK expression [33]. In chronic heart failure, the adrenal chromaffin cell results in increased GRK2 levels, which in turn results in increased phosphorylation and desensitization of alpha2ARs and subsequent increased catecholamine secretion and, thus, circulating catecholamines. The exposure to high levels of circulating catecholamines has been reported to be toxic to cardiac myocytes [34, 35] and perhaps may adversely affect the brain as well. Simultaneous inhibition of GRK2 in the heart, the adrenal gland, and brain with an appropriate pharmacological inhibitor may have a positive use in treating chronic heart failure and perhaps AD. 

G protein-coupled receptor desensitization is emerging as an important feature of several cardiovascular diseases. GRK2 plays a key role in the regulation of a variety of these receptors and cardiac muscle expression is altered in pathological situations at the promoter level such as in CHF [36], portal hypertension [37], and other tissues and cells, such as lymphocytes [38] in these conditions. GRK-dependent receptor desensitization and regulation of *β*AR or other GPCRs is a rapid process that appears to involve agonist-promoted receptor phosphorylation by GRKs. GRK-mediated receptor phosphorylation promotes the binding of arrestin proteins, such as *β*-arrestin [39]. *β*-arrestin binding uncouples G protein-coupled receptors from their respective G proteins by sterically blocking receptor coupling to G proteins. These same regulatory proteins also modulate GPCR endocytosis and the processes of transient receptor internalization, intracellular trafficking, and resensitization [40]. Further, the internalization process leads to ERK activation, as is the case for beta2AR and lysophosphatidic acid receptor [41] and ERK activation, known as a prominent feature of AD. Consequently, the *β*-arrestins play a crucial role in regulating the responsiveness of many GPCRs. GRK2, along with *β*-arrestin, plays a key role in resensitizing GPCRs by allowing its dephosphorylation and recycling. Data by Mayor and colleagues indicate that besides the uncoupling function of *β*-arrestin, which together with GRK, directly participates in beta2AR sequestration and may provide one trigger for resensitization [19]. GRK2 levels in myocardium and lymphocytes may be associated with *β*-AR dysfunction and heart failure severity. 

Signaling through cardiac betaARs is significantly impaired in many cardiovascular disorders, including CHF. Recent studies in several different mouse models have demonstrated that betaARK1 plays a key role not only in the regulation of myocardial signaling, but also in cardiac function and development [24]. Moreover, studies have shown that targeting the activity of GRKs, especially betaARK1, appears to be a novel therapeutic strategy for the treatment of the failing heart. The development of small molecule inhibitors of betaARK1 and GRK activity may advance therapeutic options for heart disease [42], which may be useful for AD as well, perhaps under conditions where excitotoxicity is evident.

## 4. GRK, ET-1, and Insulin Signaling

Diabetes and Alzheimer's disease have merged as a comorbidity factors and failures of energy homeostasis are involved in both of disease processes, as diabetic individuals have a 30- to 65-percent higher risk of developing Alzheimer disease compared to nondiabetic individuals [43, 44]. Hyperglycemia increases DAG activates PKC-*β* and -*δ*, which can decrease eNOS, increase ET-1, and effect blood flow. It also activates VEGF, which affects vascular permeability, angiogenesis, and NADPH oxidases, which increase oxidative stress. GRK2 receptor is regulating signaling at the G protein level by interacting with G*α*q, G*α*11, and G*α*14, but not G*α*s, G*α*i, G*α*12/13, or G*α*16 [45, 46]. The phosphorylation-independent binding of the N-terminal domain of GRK2 to G*α*q/11 can attenuate G*α*q/11-mediated receptor signaling of angiotensin II, endothelin receptor, thromboxane A2 receptor, thyrotropin receptors, and mGluR1a/5. This process depends on the GRK2 RGS homology domain [47, 48]. Importantly, direct activation of AMP-activated protein kinase stimulates nitric-oxide synthesis in human aortic endothelial cells. Phosphoinositide 3-kinase-dependent activation of the 5′-AMP-activated kinase (AMPK) has been demonstrated through peroxynitrite- (ONOO-) and hypoxia-reoxygenation in cultured endothelial cells. Exposure of aortic endothelial cells to ONOO- increased the phosphorylation of AMPK and its downstream enzyme endothelial nitric-oxide synthase and is accompanied by increased phosphorylation of protein kinase Czeta (PKCzeta) [49]. 

In regard to energy metabolism and homeostasis, G*α*s can activate adenylyl cyclases, converting ATP into cAMP and initiating PKA activation [50, 51]. Conversely, adenylyl cyclases are inhibited by G*α*i and the G*α*q subunits that activate phospholipase C (PLC)*β*, which hydrolyses phosphoinositol phosphate (PIP2) into diacylglycerol (DAG) and inositol 1,4,5-trisphosphate (IP3), which then activate PKC and the release of intracellular calcium. Adiponectin and its receptors are integral players in mechanisms of energy homeostasis, which stimulate glucose utilization and fatty-acid oxidation by activating AMP-activated protein kinase [52], which regulates energy homeostasis and glucose and lipid metabolism in adipocytes found in the brain. Adiponectin is relevant, because it and its receptors have recently been localized in the human pituitary gland, hypothalamus and other brain regions, and hormone producing cells, such as FSH, GH, LH, TSH, and ACTH [53]. 

When GRK2 was overexpressed in vitro, binding to PI3 kinase was found to promote PI3 kinase recruitment to the plasma membrane, increasing receptor endocytosis [54]. PI3 kinases, with catalytic and regulatory subunits, generate lipid products, which activate Akt. Class IB of PI3 kinases sole member (PI3 kinase-*γ*) is composed of the regulatory p101 subunit and the catalytic p110*γ* subunit. Upon GPCR stimulation, G*β* *γ* subunits directly bind to p110*γ* in the activation of PI3 kinase-*γ* [55]. In addition, activated G*β* *γ* subunits control the activation of several effectors, including K+ channels, small G proteins, phosphoinositide-3 kinases (PI3 kinases), and PLC*β* [56].

GRK2 interacts with components of the PI3 kinase-Akt and MAPK signaling cascades with consequences for receptor signaling and desensitization. It has also been reported that an interaction between GRK2 and Akt inhibits the kinase activity of Akt in sinusoidal endothelial cells from portal hypertensive rats [37]. PKC, PKA, and PLC*β* can mediate upstream activation of MAPKKK by G protein interaction. The MAPK cascades are MAPK kinase (MAPKKK), which phosphorylates and activates a MAPK kinase (MAPKK) and, in turn, activates one of the MAPKs, whose members are ERK1/2, JNK1-3, p38MAPK*α*/*β*/*γ*, and the more recently identified ERK5 [57]. All G*α* and G*β* *γ* subunits have been described to trigger activation of the different MAPK pathways, with some G*α* subunits exerting inhibitory effects on MAPKs [58, 59]. For example, phosphorylation of p38MAPK by GRK2 uncovers a novel mechanism of inhibition of the association of p38 with some of its partners [60].

Since we have hypothesized the existence of an imbalance between the NOS species and ET-1, we now suggest a putative role of GRK2 in chronic ET-1-induced insulin resistance in the brain and vascular wall cells, as it has been found for other cells and tissues, which also may contribute to consequences for Alzheimer and stroke patients by similar mechanisms. In that regard, GRKs, which are classical serine/threonine kinases that desensitize agonist-occupied GPCRs, have been found to regulate other receptors such as the insulin receptor (IR), which is a tyrosine kinase receptor. GRK2 was found to negatively regulate glycogen synthesis in mouse liver FL83B cells [61]. This group demonstrated that the IR also couples to G proteins, specifically GRK2, and utilizes downstream signaling components to negatively regulate IR signaling in those cells. In other tissues and cells, GRK2 can function as a negative regulator of insulin action by interfering with G protein-q/11 alpha-subunit (G*α* *q*/11) signaling [47], causing decreased glucose transporter 4 (GLUT4) translocation [48]. This same group reported that chronic endothelin-1 (ET-1) treatment leads to heterologous desensitization of insulin signaling with decreased tyrosine phosphorylation of insulin receptor substrate (IRS)-1 and G*α* *q*/11 and decreased insulin-stimulated glucose transport in 3T3-L1 adipocytes. It is GRK2 which mediates endothelin-1-induced insulin resistance via the inhibition of both G*α* *q*/11 and insulin receptor substrate-1 pathways in these cells. Another mechanism is one where GRK2 functions as a negative regulator of insulin action through cdc42-associated phosphatidylinositol 3-kinase activity and the phosphorylation of IRS-1 and IRS-1 protein degradation. Taken together, the importance of GRK2 in AD, vascular dementia, and other metabolic diseases, such as diabetes, should not be underestimated. GRK2 deficiency was found to increase the basal and insulin-stimulated phosphorylation of Ser(21) in glycogen synthase kinase-3alpha. Insulin-induced tyrosine phosphorylation of the IR was similar in control and GRK2-deficient cells [61]. Of interest here is the finding that GRK2 mediates endothelin-1-induced insulin resistance via the inhibition of both G*α* *q*/11 and insulin receptor substrate-1 pathways in 3T3-L1 adipocytes [48]. Further, the same group showed that chronic endothelin-1 (ET-1) treatment leads to heterologous desensitization of insulin signaling with decreased tyrosine phosphorylation of insulin receptor substrate (IRS)-1 and Galphaq/11 and decreased insulin-stimulated glucose transport in 3T3-L1 adipocytes. In the current study, we have investigated the role of GRK2 in chronic ET-1-induced insulin resistance.

Recent data suggest possible alternate roles for GRK2 other than as a kinase. In that regard, the role of phosphorylation of the endothelin B receptor (ETBR) in agonist-induced desensitization was investigated, using mutant lacking c-terminal 40 amino acids (delta 40 ETBR). In cells expressing the wild-type or delta 40 ETBR, ET-1 caused rapid desensitization of calcium responses [62]. These investigators found that the wild type-ETBR was phosphorylated by ET-1, and the phosphorylation was markedly enhanced when coexpressed with GRK2. However, delta 40 ETBR was not phosphorylated regardless of coexpression with GRK2. Phosphatidylinositol 3 formation was ET-1 induced in these cells and was decreased by coexpression with GRK2 or kinase-dead GRK2 by a similar mechanism, by which the authors suggest the presence of phosphorylation-independent desensitization mechanism in delta 40 ETBR as a possible alternate role for GRK2, other than those that are kinase related in the strict sense.

Of importance in these GRK2 AD studies is the cellular response to certain stimuli from the vascular endothelium, neurons, and glia, which all are able to synthesize, store, and release ROS, NO, and ET-1, a vasoactive peptide. In that regard, elevated circulating glucose concentration leads to many forms of pathology including increasing superoxide formation that can form hydrogen peroxide, leading to hydroxyl radical production, which can interact with NO to form peroxynitrite. GRK2 is impaired by hydrogen peroxide [63]. All of these reactive species can damage nucleic acids, protein, and lipids, leading to oxidative stress through loss of reducing equivalents, DNA strand breaks, poly(ADP)ribose polymerase activation, and consumption of NAD+, which ultimately impairs energy homeostasis. In support of this notion, it is important to consider PARP activation in diabetes, which is linked of increased oxidative stress and leads to NAD+ depletion [64].

 Their contribution to the pathophysiology of stroke or stroke-like conditions and AD cannot be understated. ET-1 is produced by multiple cells and is differentially coupled to G proteins [45] in response to hypertrophic stimuli in vitro and in the development of heart failure in vivo [65, 66]. Nevertheless, the endothelin A and B receptors (ET_A_-R and ET_B_-R) undergo desensitization, most likely also through GRK2 [67]. For example, ET-1 can elicit several responses; it activates EC NOS via G protein beta/gamma subunits signaling through protein kinase B/Akt [68] as well as prolonged physiologic responses, including mitogen-activated protein kinase (MAPK) activation [69] and c-Jun NH2-terminal kinase (JNK) and extracellular signal-regulated kinase (ERK) in cultured animal cells and in vivo [70]. MAP kinases have been long associated with AD and ERK activation may be another important early event, perhaps downstream from GRK2 activation [71]. Interestingly, these pathways also have been implicated in cell-cycle dysregulation in human AD cases [72, 73]. Recently we have demonstrated that since successful dysregulation of the cell cycle is also the hallmark of a neoplastic changes, early cell-cycle pathophysiology in AD may recruit oncogenic signal transduction mechanisms and, hence, could be viewed as pseudoneoplastic transformation, which is eventually aborted [74]. Further, it has been shown that phosphorylation of GRK2 by MAPK also triggers a turnover of GRK2 degradation through the proteasome pathway, in which GRK2 is targeted for proteolysis by *β*-arrestin function and Src-mediated phosphorylation [75]. Therefore, GRK2 may play a very important role in AD pathogenesis mechanisms through oxidative stress and mitochondrial dysfunction. 

## 5. GRK Studies in AD and CBH

Studies of the details and consequences of GRK's mechanisms have focused heavily on the original beta-adrenoreceptor kinase (beta-ARK) family (GRK2 and GRK3) and, in particular, on phosphorylation-dependent recruitment of adaptor proteins such as the beta-arrestins. Several lines of evidence implicate GRK and beta-arrestin expression in AD and after cerebral hypoxia/ischemia (HI) [18] and the differential GRK2 expression in compensated hypertrophy and heart failure after myocardial infarction in the rat. Moreover, G protein-coupled receptor kinases regulate metabotropic glutamate receptor 5 function and expression [76], which has also been implicated in AD pathogenesis and GRKs may offer a mechanism for desensitization of this receptor isoform.

The main experimental goal of our previous study was to investigate and better clarify the relationship between GRK2, vascular lesions, and the development of pathology in a CBH model and AD, at the cellular and subcellular levels [8]. In that regard, we examined a connection between vascular damage and predisposing factors for AD, where we explored the changes in brain distribution of GRK2 in microvessel wall cells and neurons using a CBH model and in AD cases. Our previous studies and those of others have reported that CBH will result in a 22%–30% reduction of hippocampal blood flow that will stabilize after several weeks without further reduction [77–79]. This model is relevant to examining the physiopathology of AD and stroke and enables exploration of the relationship between vascular events and AD. 

**Figure 1:**
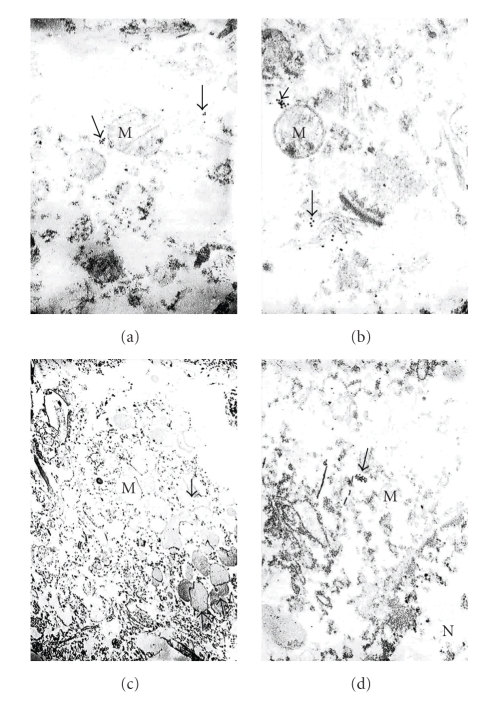
Subcellular localization of GRK2 immunoreactivity detected by using preembedding immunogold decoration in hippocampus of age-matched control (a, b) and AD brain (c, d). (a) and (b) The neuronal cell body from the age-matched control brain hippocampal tissue shows the presence of GRK2 containing gold particles (arrows) attached to the external membrane of partially damaged mitochondria. GRK2 immunopositive gold particles localized in the matrix of damaged mitochondria and Golgi cistern, X 30,000 and X 40,000, respectively, (a) and (b). (c) Hippocampal tissue from the AD brain shows that the neuronal cell body is characterized by the presence of large number of mitochondria-derived lysosomes (M) and disperse distribution of GRK2 positive gold particles (arrows), X 6,000. (d) Glial cell body from the AD brain tissue shows clusters of GRK2 immunoreactivity in the matrix of mitochondria derived-lysosomes (single arrow), X 20,000. Abbreviations: M Mitochondria; N cell nucleus, [reprinted with permission of Neurotoxicity Research [8]

**Figure 2:**
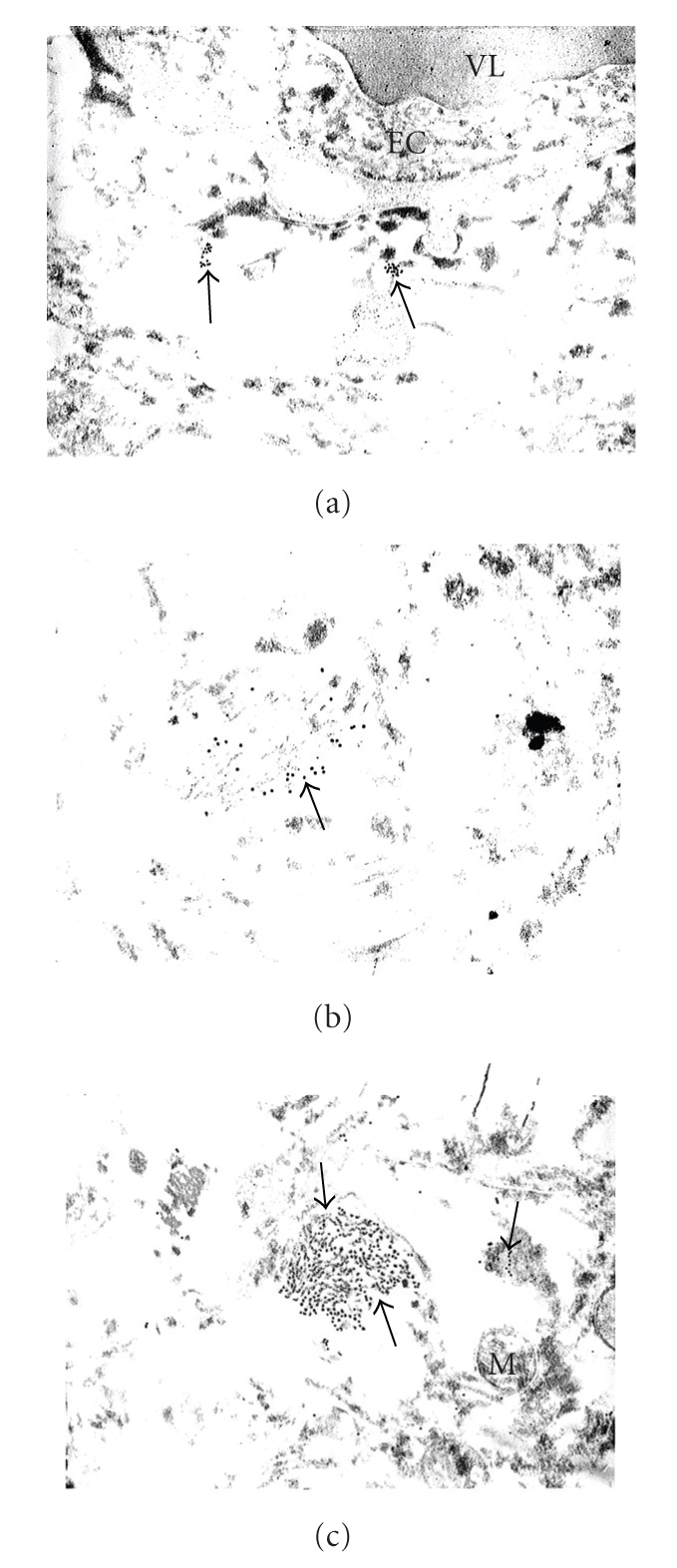
The ultrastructural localization of GRK2 immunopositive gold particles in postmortem human AD (a) and age-matched control brain (b, c) tissues. (a) The GRK2: immunopositive containing gold particles in the matrix of perivascular pericytes (indicated by single thick arrows) but not in the cytoplasmic matrix of severely damaged vascular endothelium (EC), X 40,000. (b) and (c) The neurons close to perivascular regions show the presence of GRK2 containing gold particles in their matrix, where most gold particles were associated with the neurofibrillary tangle- (NFT-) like structures (arrows). However, the intact mitochondria (M) were free from GRK2-immunopositive gold particles, X 40,000, respectively, (b) and (c). (reprinted with permission of Neurotoxicity Research [8]).

**Figure 3:**
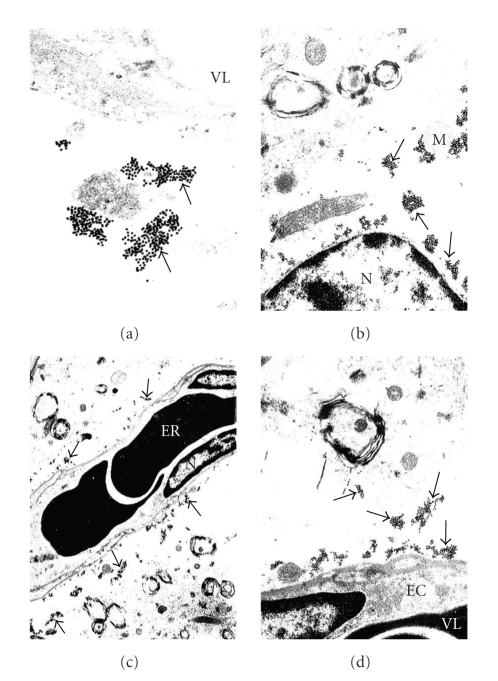
Ultrastructural characteristics of GRK2: immunopositive gold particles from the rat brain in control (same-operated: a, b) and 2-vessel occlusion (2-VO; resp., c, d) experiments. (a) Clusters of GRK2: immunopositive gold particles in the cytoplasmic matrix of perivascular pericytes (arrows) but not in the vascular EC, X 20,000. (b) The presence of GRK2 immunopositive gold particles associated with the edematous portion of the perivascular pericytes cytoplasmic matrix (arrows). Intact, but not giant, mitochondria (M) are free from any GRK2-immunopositive positive gold particles, X 30,000. (c) The GRK2 containing positive gold particles was seen in the hippocampal tissues from rat exposed to 2-VO. The presence of GRK2 immunopositive gold particles was seen throughout the matrix of damaged perivascular pericytes (arrows), X 8,000. (d) Perivascular regions of this area from figure (c) under higher magnification display the presence of islands of GRK2-containing gold particles which are associates with the damaged regions of the cytoplasmic matrix (arrows). Nucleus (N) and intact mitochondria are from the GRK2 immunoreactivity, X 30,000. (reprinted with permission of Neurotoxicity Research [8]).

**Figure 4:**
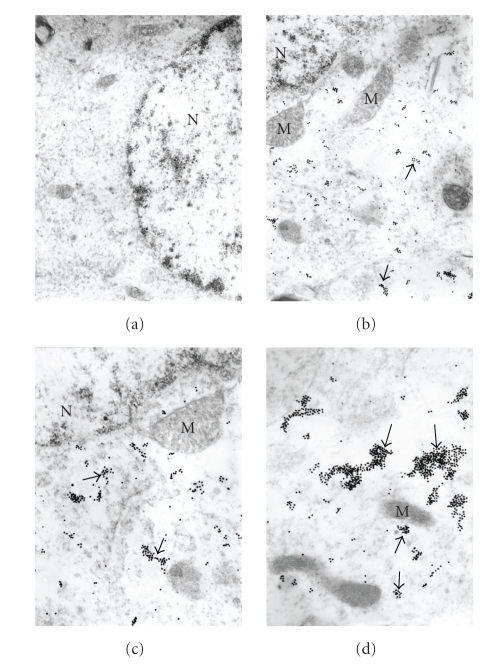
The subcellular features of the GRK2 immunoreactivity in the hippocampus of the rat subjected to 2-vessel occlusion. (a) Intact neurons show absence of any GRK2 immunopositive gold particles in their cytoplasmic matrix, X 15,000. (b) Neurons with the effect of chronic cellular hypoperfusion demonstrate the presence of a GRK2 overexpression (arrows) throughout the cell body, however, the intact mitochondria (M) were free from any GRK2 immunopositive gold particles, X 30,000. (c) *“Hypoperfusion”*-affected neuronal cell body show the presence of islands of GRK2 positive immunodecoration in the external membrane and in the matrix of damaged mitochondria and mitochondria-derived lysosomal structures (arrows), X 40,000. (d) Neurons with severe damage shows the presence of islands of GRK2 containing immunopositive gold particles that associated with the completely damaged (mitochondria-derived lysosomal structures) (arrows), but not with nondamaged mitochondria (intact and giant), X 40,000. (reprinted with permission of Neurotoxicity Research [8]).

**Figure 5:**
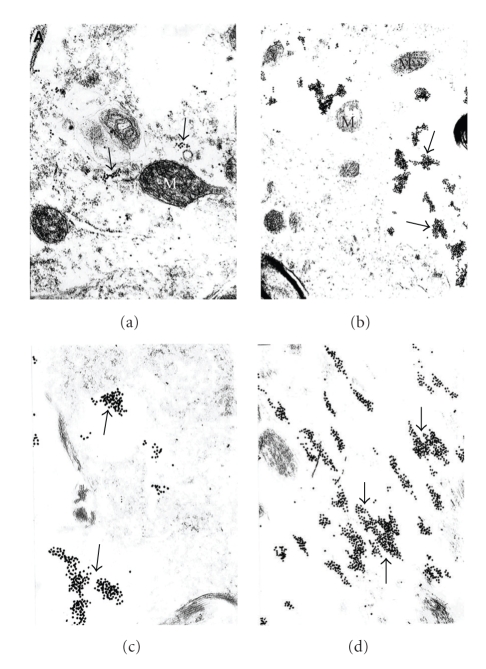
The GRK2 immunoreactivity in rat brain hippocampal tissues was exposed to 2-vessel occlusion and determined by using preembedding immunogold cytochemistry techniques. (a) Subcellular determination of GRK2 in the neuronal cell body shows the presence of GRK2 immunopositive gold particles (arrows), which associates with the external membrane and the matrix of damaged but not intact mitochondria (M), X 40,000. (b) Neurons containing granular vacuolar degenerative structures show island of GRK2 immunopositive gold particles (single arrow), X 30,000. (c) The glial cell body shows overexpression of GRK2 immunoreactivity in the matrix of granular vacuolar degenerative structures (single arrow), X 50,000. (d) Neurofilament from the damaged neurons shows the presence of GRK2 immunopositive gold particles (single arrows), X 40,000 (reprinted with permission of Neurotoxicity Research [8]).

Our study was the first where ultrastructural localization demonstrated that the overexpression of GRK2 occurs during the early stages of damage in aged human and AD cases (see Figures 1 and 2), and also in our 2-VO model of CBH (see Figures 3, 4, and 5). This overexpression is an early event, occurring at prodromal stages, before and up to a point when the damage is reversible. Based on our observations, we were able to detect the subcellular localization of GRK2 immunoreactivity in the neuronal cell body of the age-matched control brain hippocampal tissues. We achieved this by using preembedding immunogold decoration in the hippocampus of age-matched control and AD rat brains. These brains showed the presence of GRK2 containing gold particles attached to the external membrane of partially damaged mitochondria. In addition, GRK2 immunopositive gold particles localized in the matrix of damaged mitochondria and Golgi cistern. However these observations were seen in the case when aged-matched damage occurred in the hippocampal neurons. Contrary to this observation, hippocampal tissue from the AD brain showed that the neuronal cell body was characterized by the presence of large number of mitochondria-derived lysosomes and disperse distribution of GRK2 positive gold particles. Moreover, glial cell body from the AD brain tissue also shows clusters of GRK2 immunoreactivity in the matrix of mitochondria-derived lysosomes (for the details see Figure 1). In our AD and hypoperfusion studies, we observed less positive signals for GRK in control cases, generally. Mostly GRK positive gold label in electron microscopic studies was observed bound to the residues of the different cellular compartments, such as damaged mitochondria, distorted perivascular cells. Some positive signals were observed in perivascular cells associated with damaged vessels as well as in cellular compartments with lesions. Nevertheless, there are pathological cellular structures, such as NFT-like and/or vacuolar degenerative structures (GVD), which colocalized with GRK2 [8]. In some cases GRK positive signals were bound to degenerated vacuolar structures. These data were the first known in vivo evidence demonstrating GRK2 activation in early cerebrovascular disease, including AD, and thus GRK2 could serve as a new target for treatment approaches to AD, cerebrovascular dementia, or stroke [80].

Usually, GRK2 immunoreactivity was found to be associated with damaged cellular compartments, especially mitochondria and/or mitochondria-derived lysosomes or granular/vacuolar degenerative structures (see Figures 1 and 2). The immunopositive reactivity was observed in damaged vessel wall cells and their subcellular compartments (Figures 1 and 2). We have found that neurons that contain neurofibrillary tangles (NFTs) show abundant GRK2 immunopositive reactivity (Figure 2). The intensity of the reaction varied from cell to cell and within cellular compartments as well (Figures 1 and 2). However, cellular lipofuscin was not associated with any GRK2 immunoreactivity. Nevertheless, there are pathological hallmarks of AD present in harvested neurons, for example, neuronal inclusions, or those neurons containing structures such as NFTs, granular vacuolar degeneration (GVD), as well as in microvascular wall cells, which show a highly intense immunopositive reaction. While late stages of damage reveal scarce GRK2 immunorectivity in areas that were previously abundant, suggest that overexpression of GRK2 can be lost or reduced, which was confirmed by western blotting [4]. Thus, this protein can serve as an earlier marker of the brain damage that typifies cerebral-vascular and/or mild cognitive impairment, human AD, and damage in an animal model that mimics AD. In addition, the overexpression of GRK2 immunoreactivity complements our earlier observation that oxidative stress-induced damage is observed in mitochondria and/or other cellular compartments before any amyloid deposition occurs [4,67]. One mechanism of regulation of GRK2 stability includes Mdm2 and the PI3K/Akt axis, orchestrated in a stimulus, cell type, or context-specific way, as well as the functional consequences of altering GRK2 expression/functionality in specific cell types and experimental models.

A parallel study reported abnormal GRKs in vitro for early stages of AD, which is associated with early amyloid beta (A*β*) accumulation in vitro and showed that subthreshold A*β* pretreatment disrupts binding of GRKs to activated GPCRs [16]. This led to reduced membrane GRK2/5, which subsequently led to retarded GPCR desensitization, prolonged GPCR signaling, and cellular supersensitivity to GPCR agonists [16]. The same group went on to report in a transgenic mouse model of AD, where the double-mutant form of APP695 is overexpressed under the regulation of a prion promoter, the overexpression of GRK2, and to a lesser extent GRK5, occurred in the cytosolic versus membrane fractions from hippocampal and cortical brain homogenates with increasing age and plaque deposition. While the in vitro observation is quite likely to occur within microglia, the increase in the overexpression of GRK2 and GRK5 in the cytosol of neurons was not differentiated in this study. Nevertheless, we report the subcellular localization of GRK2 in neurons and the earlier involvement of vascular lesions in vivo as a key event in this process and, thus, in the development of human AD and AD-like pathology. Data to support this notion have been explored in various rat models [77, 81]. In this regard, we have demonstrated that abnormal mitochondria (mitochondria with electron dense matrix and mitochondrial-derived lysosomes) and lipofuscin appear to be features of damaged hippocampal neurons in aged Tg (+) mice and human AD, suggesting a direct relationship between vascular abnormalities, BBB breakdown, neuronal loss, and amyloid deposition [4, 10, 12, 14, 82]. 

Our in vivo data were discussed in light of another study involving amyloid [16]; our model shows a similar effect, but attributes the overexpression of GRK2 to oxidative stress and events prior to A*β* deposition. While the effect observed in the Sou study involved subthreshold levels of the protein, which may reflect an early event as well, the use of total homogenates from the transgenic model of A*β* overexpression does not indicate which cells are affected, failing to control for glia or other immunologic cells that may be involved. However, since A*β* deposition is a later hallmark lesion in AD, we suspect that the appearance of A*β* along with the loss of GRK2 immunoreactivity may be linked somehow, but the role of A*β* on GRK2 translocation may be cell specific and has not been characterized. Therefore, the appearance of A*β* is unlikely to be the primary predisposing factor for GRK2 overexpression, as A*β* deposition occurs much later in the disease process. Any cytotoxicity may emanate from mechanisms other than amyloid directly as earlier events seem to be more crucial in the disease pathogenesis. Perhaps early cytotoxicity resulting from non-amyloid-mediated mechanisms may be more crucial in the etiopathology and suggest that A*β* is less likely to be the primary predisposing factor for GRK2 overexpression [12, 83]. 

Our studies demonstrate an increase in GRK2 localization to the cytosol, but in particular to subcellular components and only those components with damage and/or pathology evident (see Figures 1–5). In our study we used perfusion fixation for the 2-VO model and for this reason preservation of the tissue in 2-VO exposed animals was much better than when compared to human postmortem AD brain tissues, even after a very short postmortem time period (<2–4 hours). However, in the general distribution and the density of the GRK-2 containing gold particles, it was very similar in the human AD and 2-VO animal models. Recruitment of GRK to the cell membrane is followed by inhibition of signaling. Therefore, the sequestration of GRKs to subcellular locations may indicate a compensatory adaptation to AD. However, other studies suggest that GRKs have more diverse protein/protein cellular interactions and that *β*-arrestins together with GRKs play a crucial role in regulating the responsiveness of many GPCRs. Further, GRK2 levels in myocardium and lymphocytes may be associated with *β*-AR dysfunction as well, which is one area that should be addressed in AD. One explanation for the subsequent loss of GRK2 may lie in the ability of A*β* to act as a bioflocculant [84] and a possible role in the sequestration of GRK2, thereby limiting downstream phosphorylation events as well, or leading to translocation of GRK2 to the cytosol. Regardless, the reduced availability of GRK2 and beta-arrestins to regulate GPRC signaling most likely would lead to a state of GPCR supersensitization, thereby increasing response to neuropeptides, neurotransmitters, chemokines, and many other molecules; all of which could have deleterious consequences. Conversely, it may be plausible that increased GRK2 expression, and particularly localization of an expression of modulation, and this would impart a compensatory or survival response to excitotoxicity, which is a claim made for A*β* as well [85]. Finally, neurodegeneration can have numerous overlapping features, and GRK2 along with the action of specific phosphatases has been implicated in other neurodegenerative diseases such as amyotrophic lateral sclerosis (ALS) [86]. Thus, there are numerous parallels that can be drawn between the neurodegenerative and cerebrovascular disorders with heart disease and systemic vascular disorders, which drives home the important connection the role GRK can have in all disorders, particularly AD [87]. 

In comparison to controls, ultrastructure in AD and animal models are predominated by abnormal mitochondria. Studies examining deleted mtDNA and mitochondrial-derived lysosomes in regions closely associated with lipofuscin suggest that proliferation, deletion, and duplication of mtDNA occur in mitochondria in human AD and transgenic mouse models of neurodegeneration [4, 10, 12, 14, 82]. In situ hybridization with a chimeric mouse and human mitochondrial cDNA probes for the 5 kb common deletion indicate that the deletion is increased at least 3-fold in AD cases as compared to controls and in yeast artificial chromosome (YAC) APP mouse hippocampus [4, 10, 14, 88], which is strongly positively correlated (*r* = 0.934) with the marker of DNA oxidation, 8-OH deoxyguanosine. These findings indicate that the mitochondrial DNA overproliferation and/or deletion are key initiating factors for disruption of the BBB and the development of pathology, and GRK2 immunoreactivity overexpression would be coincident with these processes. 

Earlier in a 2-VO model, we reported that ultrastructural examination of hippocampal CA1 capillaries in rats revealed a smaller-size EC containing damaged mitochondria, characterized by transformation of lysosomal structures within the EC and in the perivascular area [83]. Along with mitochondrial abnormalities these changes appeared to be associated with amyloid deposition found surrounding the capillary vessel wall. Electron microscopic immunoassaying showed sparse endothelial-specific NOS- (eNOS-) containing positive gold particles in the matrix of the vascular endothelium, in contrast to substantial labeling in the cytoplasmic matrix of perivascular cells and electron-dense mitochondria, indicative of a hypoxic insult [83]. Immunoreactive eNOS-containing positive gold particles were found markedly expressed in hippocampal neurons and in glial cells, when compared to nonoccluded controls [83]. Of interest is the comparison between the eNOS overexpression pattern to that of GRK2 [37], which has the same pattern as that of our previous observation of eNOS. 

The EM findings in rat hippocampus after CBH also support the general hypothesis that chronic oxidative stress caused the EC structural changes and the mitochondrial and immunoreactive eNOS changes, since such changes were observed only in 2-VO rats. In addition, previously we have demonstrated that oxidative damage is the earlier event in AD [89]. These findings support our working hypothesis that oxidative stress-induced vascular changes, such as an abnormality in vascular NO, are important molecules in spatial memory functions, at least in this CBH model. Further, this damage coexists with overexpression of GRK2 immunoreactivity and in rats, hypoxia/ischemia modulated GRK2, and beta-arrestin-1 levels [90].

**Figure 6:**
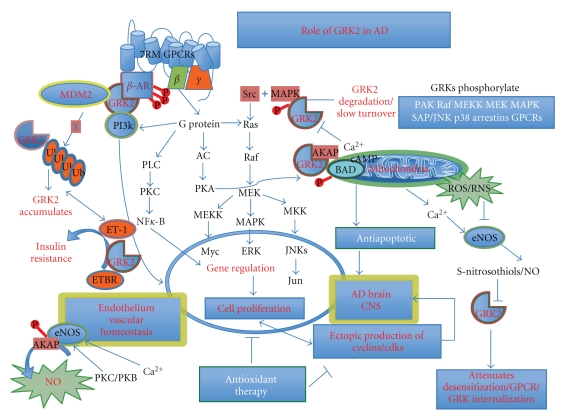
The role of GRK2 in AD showing the activation pathways and consequences of increased expression of GRK2 with the endothelium, brain, and mitochondria.

 Therefore, chronic oxidative stress-mediated inhibition of eNOS may coexist with the early overexpression of GRK2 immunoreactivity and would appear to support a compensatory role or reaction in brain tissue to potentially mitigate chronic injury stimuli such as oxygen depletion and nutrient deficiency or imbalance in metabolic homeostasis found in 2-VO conditions [83]. These data support the present observation that it is chronic injury stimuli that not only initiates damage and compensatory changes but also accelerates brain damage in tissues, which can contribute to some types of mental retardation and cognition deficits involving the consequence of A*β* accumulation in the brain and in some types of mental retardation, such as Down Syndrome [91]. The connection to a cerebrovascular component to AD is further borne out in other rat studies, where differentially expressed cardiac GRK2 expression and activity have been found. Here, GRK2 expression has been reported to be inhibited in animals with cardiac hypertrophy without heart failure, whereas animals with heart failure had elevated GRK2 [18]. The same can be said for failing human hearts, which are reported to have elevated levels of GRK2 [92]. This expression pattern indicates differential regulation in hypertrophic nonfailing and hypertrophic failing hearts. Nevertheless, it is now a commonly held belief that GRKs may likely become effective therapeutic targets for heart disease [42] and should be considered for AD or related neuropathology as well (Figure 6).

## 6. Conclusions

Our growing understanding of GRK2 and its cognate regulatory proteins provides support for a unifying hypothesis of AD where these proteins play a pivotal role by linking the many phenomenological observations into a conceptual framework that contributes to a growing body of evidence favoring the reclassification of AD as, primarily, a cerebrovascular disorder. For example, one clue also may lie in the finding that GRK2 is a microtubule-associated protein, and tubulin was identified as a novel GRK2 substrate [93]. These results suggest that tubulin is most likely phosphorylated in situ by GRK2 and that the phosphorylation may affect the interaction of microtubules with microtubule-associated proteins (MAPs) [94]. Phosphorylation by GRKs may have downstream consequences for neuronal cell death and perhaps contribute to the hyperphosphorylated state of tau protein, as seen in AD or in earlier events as well, perhaps one that would predispose to neuronal toxicity via NFT formation. However, recent work has revealed potential phosphorylation-independent regulation of GPCRs by GRK2 and GRK3 [21] and GRK2 was not found to phosphorylate MAPs under conditions where MAPs were already well phosphorylated by endogenous kinases, which copurified with tubulin [95]. Nevertheless, the role of this kinase in early phosphorylation of tau cannot be discounted. Therefore, GRK2-mediated desensitization may involve many diverse mechanisms. However, the role of GRKs may be a pivotal one in AD pathology, as GRK-mediated desensitization, in the absence of phosphorylation and arrestin binding, has been reported for metabotropic glutamate receptor 1 (mGluR1), the gamma-aminobutyric acid B receptors [96], and in regulation of metabotropic glutamate receptor 5 function and expression [97]. Both of these receptors have been implicated in AD pathogenesis as well [98, 99]. Therefore, GRKs may hold hope as therapeutic targets for AD and related pathologies. Taken together, this line of evidence strongly supports our findings of a role for GRK2 as an earlier marker in AD pathogenesis and may couple the contribution of oxidative stress, NO, eNOS, and ET-1 to the pathobiology of AD (Figure 6).

Our findings also suggest a role for GRK2 as a GPCR signal transducer, which may mediate the effects of GPCR activation on cytoskeletal structure and function in AD [8]. Our study is the first to demonstrate the cellular and subcellular localizations and offer in vivo evidence for GRK2 activation as an early sign of cerebrovascular aging complications in age-associated diseases involving cerebrovascular abnormalities, neurodegeneration, and cognitive impairment before any amyloid deposition can be seen. GRKs as physiological regulators could become an appropriate target for future pharmacological intervention. Moreover, determining the mechanisms of the damage, or potential protective nature of GRK2 receptor antagonist, may provide crucial information in the development of new and more effective therapies for stroke and AD patients. Further, research in this direction may enable GRKs to serve as a new target for treatment approaches to AD, stroke, mild cognitive impairment, or related cerebrovascular disorders.
